# Skin irritation and potential antioxidant, anti-collagenase, and anti-elastase activities of edible insect extracts

**DOI:** 10.1038/s41598-021-02382-0

**Published:** 2021-11-25

**Authors:** Kankanit Yeerong, Suwannee Sriyab, Suvimol Somwongin, Chanun Punyoyai, Panuwan Chantawannakul, Songyot Anuchapreeda, Adchara Prommaban, Wantida Chaiyana

**Affiliations:** 1 Department of Pharmaceutical Sciences, Faculty of Pharmacy, Chiang Mai University, Chiang Mai, 50200 Thailand; 2 Bee Protection Laboratory, Department of Biology, Faculty of Science, Chiang Mai University, Chiang Mai, 50200 Thailand; 3 Division of Clinical Microscopy, Department of Medical Technology, Faculty of Associated Medical Sciences, Chiang Mai University, Chiang Mai, 50200 Thailand; 4 Research Center of Pharmaceutical Nanotechnology, Faculty of Pharmacy, Chiang Mai University, Chiang Mai, 50200 Thailand; 5 Innovation Center for Holistic Health, Nutraceuticals, and Cosmeceuticals, Faculty of Pharmacy, Chiang Mai University, Chiang Mai, 50200 Thailand

**Keywords:** Ecology, Medical research

## Abstract

This study aimed to investigate antioxidant, anti-aging, and irritation properties of Thai edible insect extracts, including *Bombyx mori*, *Omphisa fuscidentalis*, *Euconocephalus* sp., *Patanga succincta*, *Acheta domesticus*, and *Lethocerus indicus*. Insects were extracted by 2 different methods, including maceration using ethanol or hexane and digestion using DI water. Then the extracts were determined for protein content using bicinchoninic acid assay and antioxidant activities using 2,2′-azinobis (3-ethylbenzothiazoline-6-sulfonic acid), 2,2-diphenyl-1-picrylhydrazyl, ferric reducing antioxidant power, and ferric thiocyanate assays. Anti-aging activities were investigated by determination of collagenase and elastase inhibitory activities using spectrophotometric assay. Maceration by hexane yielded the highest extract content, whereas aqueous extract from digestion possessed the significantly highest protein content and biological activities (*p* < 0.05). Interestingly, aqueous extracts of *A. domesticus* possessed the significantly highest biological activities (*p* < 0.05) with Trolox equivalent antioxidant capacity value of 8.8 ± 0.1 mmol Trolox/mg, DPPH^·^ inhibition of 19.5 ± 3.8%, equivalent concentration of 12.1 ± 0.7 µM FeSO_4_/mg, lipid peroxidation inhibition of 31.3 ± 2.4%, collagenase inhibition of 60.8 ± 2.1%, elastase inhibition of 17.0 ± 0.1%, and no irritation effect on chorioallantoic membrane and volunteers. Therefore, aqueous extract of *A. domesticus* would be suggested for further topical product development.

## Introduction

Insects, an arthropod in the class of Insecta and subphylum of Hexapoda, not only play an important role in the world ecology system, but also are a rich source of proteins, lipids, carbohydrates, minerals, and vitamins^1,2^. Nowadays, insects are a new source of animal-based protein that could overcome serious worldwide nutrition problems and deficiency of good quality protein for low-income people^3^. Approximately 1500–2000 species of insects are consumed by 3000 ethnic groups across 113 countries in Asia, Africa, Australia, and Latin America^1,2^. In Thailand, insects in a wide range of insect family, such as Tettigoniidae, Crambidae, Gryllidae, Bombycidae, Acrididae, Belostomatidae, etc. are consumed. Bamboo caterpillar (*Omphisa fuscidentalis*; Family: Crambidae) and silkworm pupae (*Bombyx mori*; Family: Bombycidae) are the most popular edible insects^4^. Grasshopper (*Euconocephalus* sp.; Family: Tettigoniidae), Bombay locust (*Patanga succincta*: Family: Acrididae), house cricket (*Acheta domesticus*; Family: Gryllidae), and giant water bug (*Lethocerus indicus*; Family: Belostomatidae) are also widely consumed throughout the country. The nutritional values of Thai edible insects have already been reported^4^. The *O. fuscidentalis* could provide the highest calories (231 kcal/100 g) since it contains the highest amount of fat (20.4%), whereas *P. succincta* contains a small amount of fat (4.7%) but it is rich in protein content (27.6%).

Although there have been several studies about Thai edible insects, most of them were related to their nutritional constituents. The biological activities of Thai edible insects, including antioxidant and anti-aging activities, have not been reported. This study would be the first to present antioxidant and anti-aging activities of Thai edible insect extracts, including *B. mori*, *O. fuscidentalis*, *Euconocephalus* sp., *P. succincta*, *A. domesticus*, and *L. indicus*. Additionally, the irritation properties were also firstly investigated in the present study to confirm their safety for topical application.

## Results and discussion

### Insect extracts

**Figure 1:**
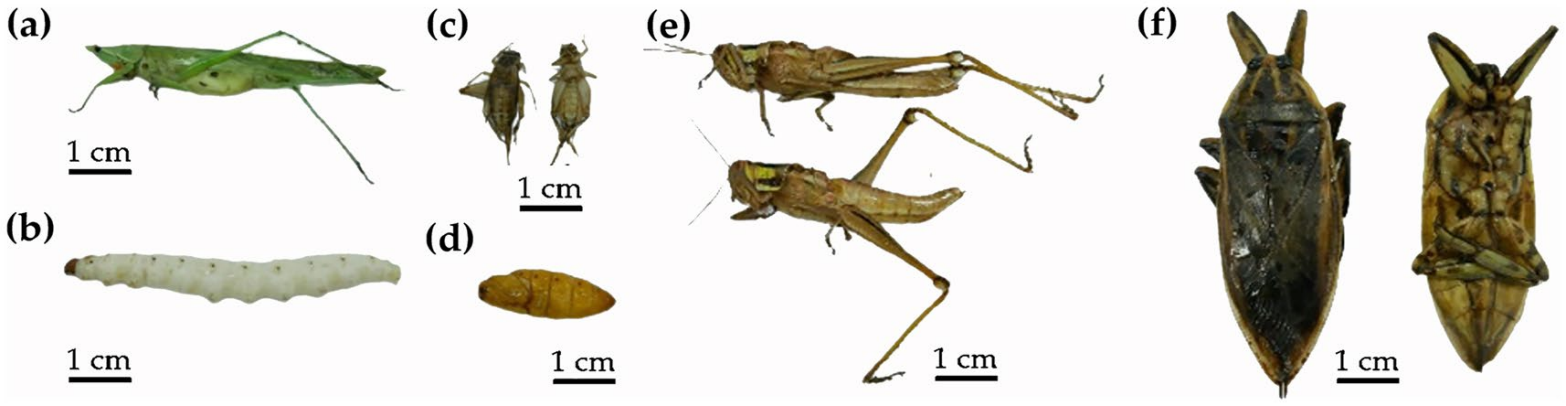
External appearances of Thai edible insects, including (**a**) rice grasshopper (*Euconocephalus* sp.), (**b**) bamboo caterpillar (*O. fuscidentalis*), (**c**) house cricket (*A. domesticus*), (**d**) silkworm pupae (*B. mori*), (**e**) Bombay locust (*P. succincta*), and (**f**) giant water bug (*L. indicus*).

**Figure 2:**
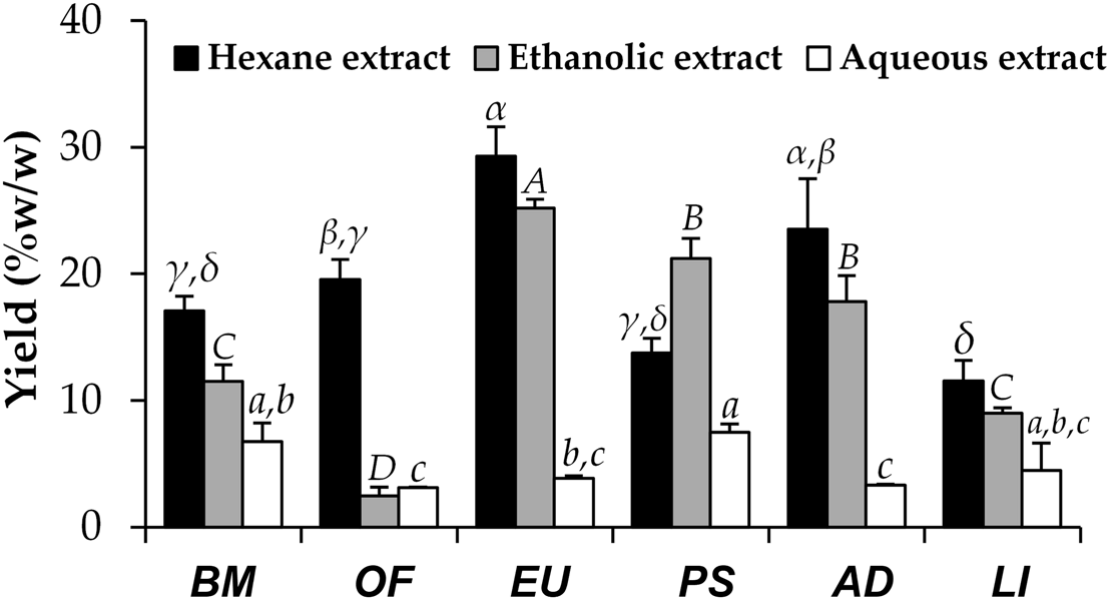
Yields of insect extracts, including *B. mori* (BM), *O. fuscidentalis* (OF), *Euconocephalus* sp. (EU), *P. succincta* (PS), *A. domesticus* (AD), and *L. indicus* (LI). The data are expressed as mean ± SD (n = 3). The Greek alphabet letters (*α, β, γ*, and *δ*) indicate significant differences among hexane extracts, the capital letters (*A, B, C,* and *D*) indicate significant differences among ethanolic extracts, and the small case letters (*a, b,* and *c*) indicate significant differences among aqueous extracts. The data were analyzed using One-Way ANOVA followed by post hoc Tukey test (*p* < 0.05).

Thai edible insects (Fig. 1) were extracted and yield of each extract is shown in Fig. 2. Hexane extracts of most insects, except for *P. succincta*, provided the highest yield, followed by ethanolic extracts, and aqueous extracts, respectively. The reason might be due to a high amount of fat content of insects. Since these fat components are hydrophobic, they could be extracted well using nonpolar solvent, e.g. hexane. Semi-polar solvent like ethanol could also be used to extract hydrophobic compounds but with less extraction efficacy^5^. Several previous studies reported that fat was abundant in biomass of insects, ranging from 4.2 to 77.2%, which was accounted for about 26.8% on average dried insects^6,7^.

Among several insect extracts, *Euconocephalus* sp. yielded the significantly highest extract content when extracted by hexane (29.3 ± 2.3% w/w) (*p* < 0.05), followed by *A. domesticus* (23.5 ± 4.0% w/w), *O. fuscidentalis* (19.6 ± 1.6% w/w), *B. mori* (17.1 ± 1.2% w/w), *P. succincta* (13.8 ± 1.2% w/w), and *L. indicus* (11.5 ± 1.7% w/w), respectively. Similarly, *Euconocephalus* sp. yielded the significantly highest extract content when extracted by ethanol (25.2 ± 0.7% w/w) (*p* < 0.05), but followed by *P. succincta* (21.2 ± 1.6% w/w), *A. domesticus* (17.8 ± 2.0% w/w), *B. mori* (11.5 ± 1.3% w/w), *L. indicus* (9.0 ± 0.5% w/w), and *O. fuscidentalis* (2.5 ± 0.7% w/w), respectively. The reason might be due to high fat content in the hexane and ethanolic extracts of *Euconocephalus* sp. The previous studies reported that fat content of some grasshopper species belonging to Acrididae family was in range of 4.2 to 22.2% of body weight^8^, whereas fat content of *A. domesticus* was around 10% of body weight^9^ and *P. succincta* contained a low amount of fat (~ 1.5% of the body weight)^7^.

In contrast, yields of aqueous extracts of all insects, which were extracted by digestion, were the lowest among various solvents. Owing to duration of extraction and numbers of extraction cycles.

### Protein content of insect extracts

**Figure 3:**
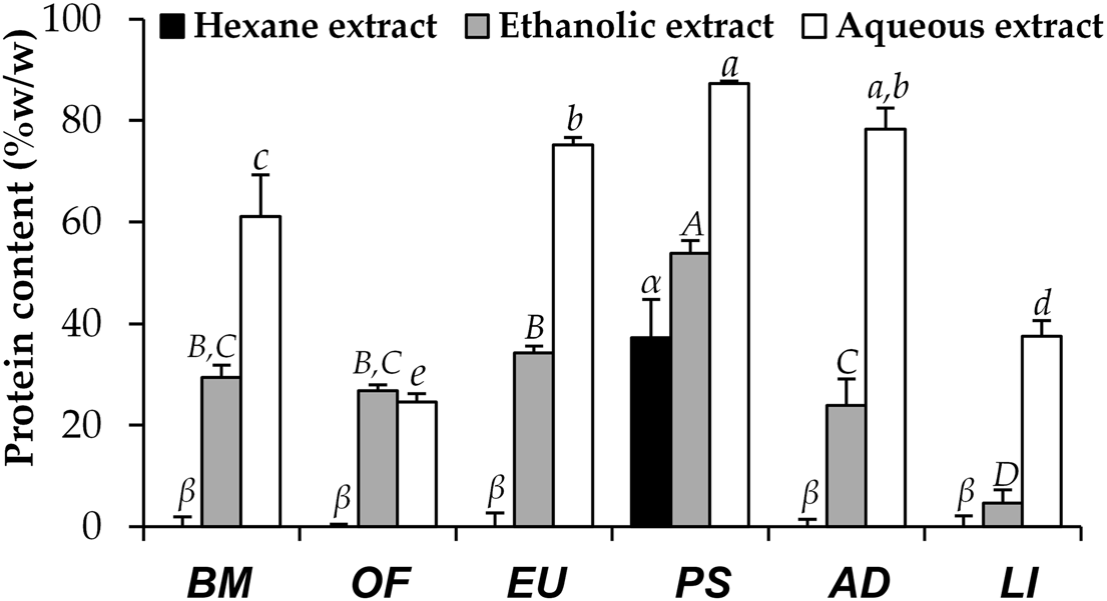
The protein content of Thai edible insect extracts, including *B. mori* (BM), *O. fuscidentalis* (OF), *Euconocephalus* sp. (EU), *P. succincta* (PS), *A. domesticus* (AD), and *L. indicus* (LI). The data are expressed as mean ± SD (n = 3). The Greek alphabet letters (*α* and *β*) indicate significant differences among hexane extracts, the capital letters (*A, B, C,* and *D*) indicate significant differences among ethanolic extracts, the small case letters (*a, b, c, d,* and *e*) indicate significant differences among aqueous extracts. The data were analyzed using One-Way ANOVA followed by post hoc Tukey test (*p* < 0.05).

The protein content of each insect extract is shown in Fig. 3. The present study demonstrated that aqueous extracts of most insects contained higher protein content than ethanolic extracts. Additionally, protein was not detected in hexane extracts of most insects. The reason was due to the less extraction efficiency of ethanol to extract protein comparing to DI water since DI water is more hydrophilic. Additionally, some protein might be denatured or precipitated during the extraction process^10^. Similarly, hexane which is non-polar organic solvent could not extract protein well because of its lipophilicity that was incompatible with protein. Apart from protein component, the compositions of hexane extract were nonpolar compounds, e.g. flavonoids, lipids, saturated fatty acids, monounsaturated fatty acids, and polyunsaturated fatty acids^11^. These results corresponded well to a previous study noted that high protein content (68.8 ± 0.4% w/w) was detected in aqueous extract of *Protaetia brevitarsis* larvae^12^.

The present study revealed that the protein content of the insects extracted by maceration and digestion ranged from 4.7 ± 2.6 to 87.3 ± 0.5% w/w which were related to a previous study reported that dry basis protein content of the insects ranged from 15 to 81%^7^. Interestingly, the aqueous, ethanolic, and hexane extracts of *P. succincta* had the significantly highest protein content among several insects, which were 87.3 ± 0.5, 53.8 ± 2.5, and 37.2 ± 7.4% w/w, respectively (*p* < 0.05). These findings were in good agreement with a previous study that reported the protein content of defatted locust extract, investigated using the Kjeldahl method, was 82.3% of dry weight, although the estimated protein content of a sample may be varied depending on the technique applied^13^. Kjeldahl is a method that involves digesting food with a strong acid, which results in the release of nitrogen, which is then measured using a titration approach^14^. Although the Kjeldahl technique is regarded as the worldwide standard and therefore simple to compare findings with other laboratories, it does not measure real protein and can result in overestimation of protein owing to the use of the standard nitrogen correction factor^14^. On the other hand, the BCA technique, which is used in the present study, is based on two chemical reactions, including the biuret reaction, which reduces cupric ions (Cu^2+^) to cuprous ions (Cu^1+^) via peptide bonds and the chelation of one Cu^1+^ molecule with two BCA molecules to form a bright purple complex which is spectrophotometrically detected^15^. Besides, BCA is simpler, ease of use, takes less time for the experiment, requires fewer instruments, high sensitivity, and tolerance of interfering species, e.g. common surfactants^15–17^. The findings revealed that the protein contents analyzed by BCA in the current investigation were equivalent to those found in the prior study utilizing the Kjeldahl method.

Therefore, protein might be a major component in most insect extracts and was efficiently extracted by water, an environmentally friendly method. However, several previous studies demonstrated that protein content of insects could be affected by different environmental factors, including origin, stage of life, and feeding^18^.

### Antioxidant activities of insect extracts

Antioxidant activities of Thai edible insect extracts were determined by 4 different methods, including ABTS, DPPH, FRAP, and FTC assay since multiple reactions and mechanisms are reported which involve antioxidant process. Both ABTS and DPPH assays indicate the abilities of test compound to scavenge free radicals and are expressed as TEAC value and DPPH^·^ inhibition percentage, respectively^19^. While FRAP assay represents antioxidant potential of test sample through the reduction of ferric iron (Fe^3+^) to ferrous iron (Fe^2+^), which is expressed as EC_1_ value. Additionally, FTC assay is the most studied biologically relevant free radical chain reaction that indicates a protective effect on lipid peroxidation of test compound^20^. Therefore, these methods were used to confirm the antioxidant activities of insect extracts.

**Figure 4:**
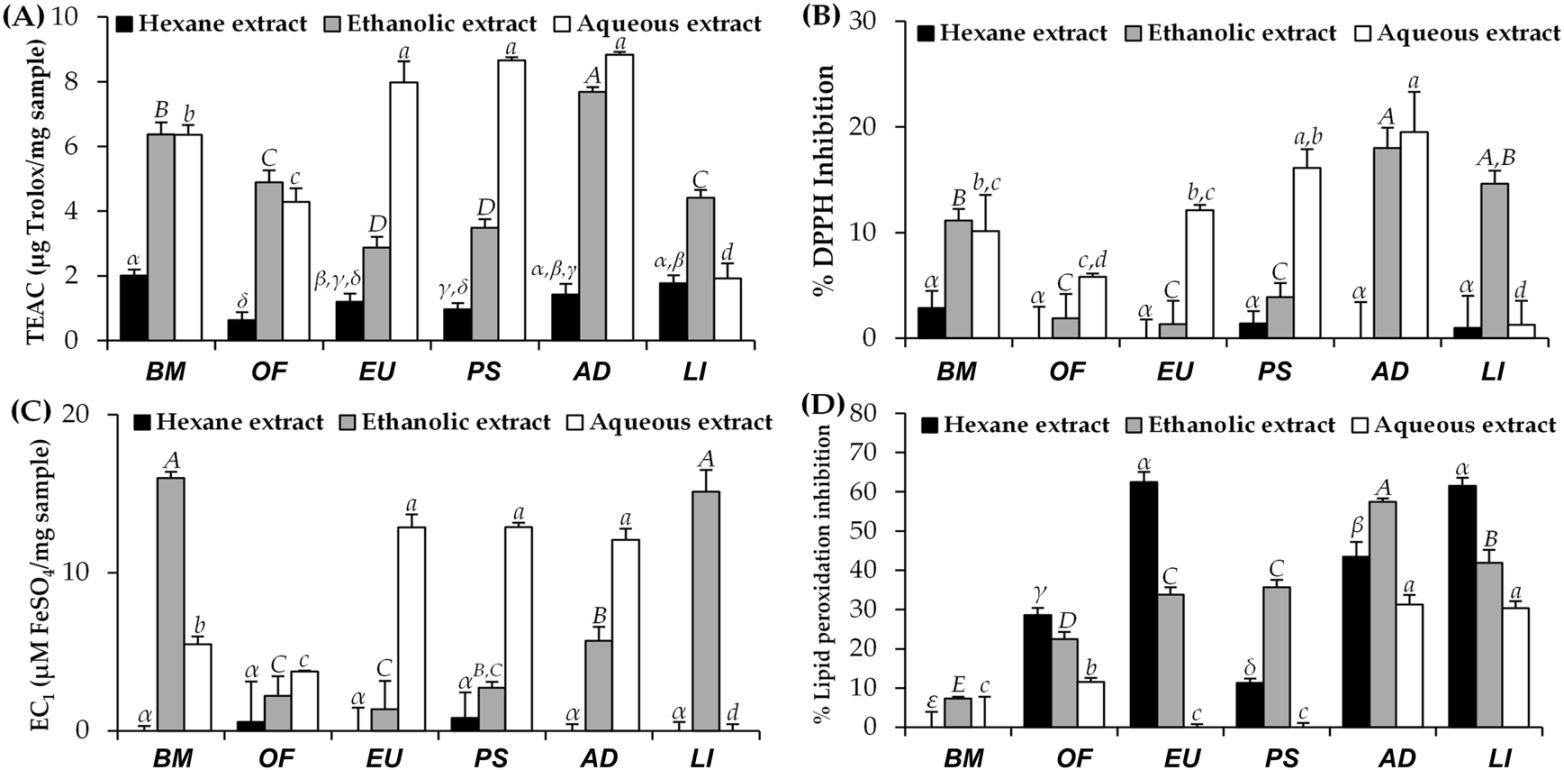
Antioxidant activities of Thai edible insect extracts, including *B. mori* (BM), *O. fuscidentalis* (OF), *Euconocephalus* sp. (EU), *P. succincta* (PS), *A. domesticus* (AD), and *L. indicus* (LI) investigated by ABTS assay (**A**), DPPH assay (**B**), FRAP assay (**C**), and FTC assay (**D**). The data are expressed as mean ± SD (n = 3). The Greek alphabet letters (*α, β, γ, δ,* and *ε*) indicate significant differences among hexane extracts, the capital letters (*A, B, C, D,* and *E*) indicate significant differences among ethanolic extracts, the small case letters (*a, b, c,* and *d*) indicate significant differences among aqueous extracts. The data were analyzed using One-Way ANOVA followed by post hoc Tukey test (*p* < 0.05).

The antioxidant activity of each insect extract is shown in Fig. 4. The hydrophilicity of extracted solvent tended to affect the antioxidant activities since both aqueous and ethanolic extracts possessed dominant antioxidant activities in ABTS, DPPH, and FRAP assay, whereas hexane extracts possessed dominant inhibitory activity against lipid peroxidation in FTC assay. The likely explanation might be due to the compatibility of test compounds with test systems^21^. Interestingly, the aqueous extracts, which were obtained from digestion for only 3 h, tended to possess higher antioxidant activities than the ethanolic extracts. The likely explanation might be due to higher hydrophilic property of aqueous (ε = 78.4) comparing to ethanol (ε = 24.5)^22^. Additionally, higher temperature used in digestion process could be another factor leading to higher extraction efficiency of aqueous. Therefore, the aqueous extracts of most insects possessed the highest scavenging activities on ABTS^·+^ and DPPH^·^, as well as ferric reducing abilities comparing to other solvents. However, *B. mori* and *L. indicus* ethanolic extracts possessed the dramatically highest ferric reducing abilities. This could be explained by various bioactive compounds which have been previous detected in *B. mori* and *L. indicus*, e.g. alkaloids, phenolic, and flavonoid compounds, which could be extracted well by ethanol^21,23^.

Among various insect extracts, *A. domesticus* and *P. succincta* aqueous extracts were predominant as radical scavenger and reductant with the significantly highest TEAC values of 8.8 ± 0.1 and 8.7 ± 0.1 μg Trolox/mg sample, DPPH^·^ scavenging activities of 19.5 ± 3.8% and 16.1 ± 1.8%, and EC_1_ values of 12.1 ± 0.7 and 12.9 ± 0.3 µM FeSO_4_/ mg sample, respectively (*p* < 0.05). These findings were in good accordance with a previous study reported that *Gryllodes sigillatus*, which is one of cricket species, possessed the highest ABTS^·+^ and DPPH^·^ scavenging activities among several insects^24^. Additionally, these results corresponded well to a previous study reported that the aqueous extracts of some grasshopper species and *A. domesticus* possessed the potent ferric reducing abilities with EC_1_ values of 2.1 ± 0.2 and 1.8 ± 0.1 mmol Fe^2+^/100 g sample^25^. Thus, the mechanisms of aqueous insect extracts on oxidation inhibition were various, including radical scavenging activities and ferric reducing abilities.

Apart from *A. domesticus* and *P. succincta* aqueous extracts, the significantly highest ferric reducing abilities were also detected in ethanolic extract of *B. mori* and *L. indicus*, with EC_1_ values of 16.0 ± 0.4 and 15.1 ± 1.4 µM FeSO_4_/mg sample, respectively (*p* < 0.05). The likely explanation might be due to several antioxidant compounds, e.g. quercetin-3,4,-O-diglucoside, phenolics, flavonoids, and riboflavin (vitamin B2), which have been detected in *B. mori* and *L. indicus*^26^. Since *B. mori* was normally fed with mulberry leaves, quercetin-3,4,-O-glucoside, a derivative of quercetin with two beta-D-glucosyl residues attached at positions 3′ and 4′ which is rich in mulberry leaves, was also detected in *B. mori*^27^. According to a potent ferric reducing ability of quercetin-3,4,-O-glucoside^28^, *B. mori* ethanolic extract also exhibited strong ferric reducing ability.

On the other hand, the results of lipid peroxidation inhibitory activities showed a different trend in comparison to the other methods. The hexane extract of *Euconocephalus* sp. and *L. indicus* had the significantly highest lipid peroxidation inhibitory activities with lipid peroxidation inhibition of 62.5 ± 2.6% and 61.6 ± 2.0%, respectively (*p* < 0.05). The reason would be lipid peroxidation test system was more compatible with hydrophobic test compounds, which were extracted well using non-polar solvent, e.g. hexane^29^.

**Figure 5:**
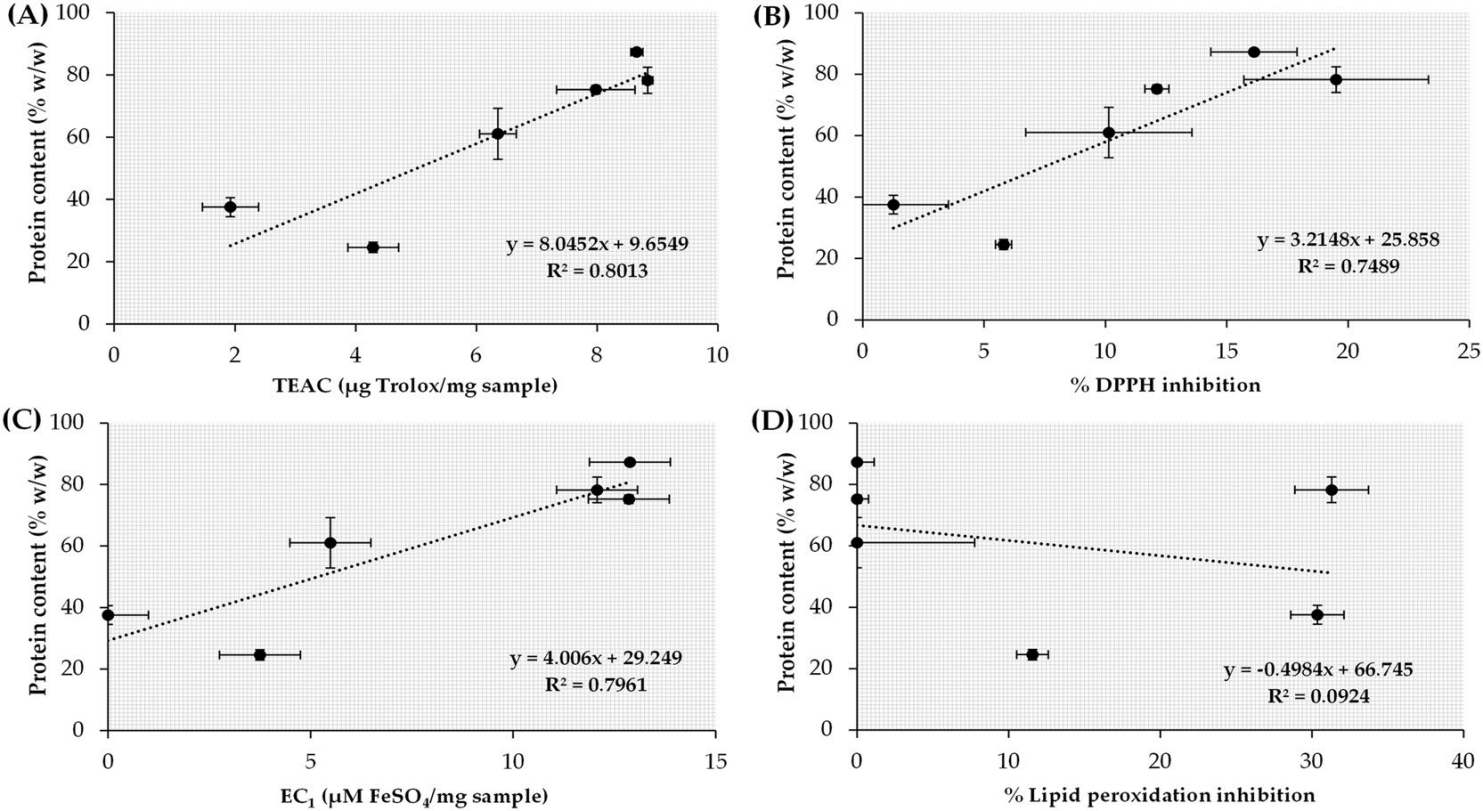
Correlations between protein content and antioxidant activities of aqueous insect extracts, investigated by ABTS assay **(A)**, DPPH assay **(B)**, FRAP assay **(C)**, and FTC assay **(D)**.

The correlations between the protein content of aqueous insect extracts and their antioxidant activities from various tests, including ABTS, DPPH, FRAP, FTC assays, are shown in Fig. 5. A strong positive correlation was detected in ABTS^·+^ scavenging activities with an R^2^ of 0.8013. Additionally, these graphs showed moderate positive correlations between the protein content of aqueous insect extracts and antioxidant activities in DPPH and FRAP assay with R^2^ of 0.7489 and 0.7961, respectively. Hence, protein from aqueous insect extracts was a major antioxidant compound that possessed radical scavenging activities and ferric reducing abilities. A previous study suggested that amino acids were found to be efficient antioxidants due to their chelating properties^30^. The amino acids had an ability to convert hydroperoxides into imines and sulfur-containing amino acids and could reduce hydroperoxides into the respective inactive hydroxylic derivatives^30^.

In contrast, there was no relationship between the protein content and inhibitory activities against lipid peroxidation (R^2^ = 0.0924). The explanation might be due to the incompatibility of protein with the lipid peroxidation inhibition test system since most of protein was hydrophilic and soluble well in polar solvent. Therefore, hexane insect extracts might contain some other bioactive compounds, which owning lipid peroxidation inhibitory activity, e.g. flavonoid, lipids, saturated fatty acids, monounsaturated fatty acids, and polyunsaturated fatty acids^11^.

### Anti-aging activities of insect extracts

Collagen and elastin are predominant extracellular matrix components presented in dermal layer of human skin^31^. Collagen fibers, which are produced by fibroblasts, are responsible for tensile strength and toughness of skin. These fibers can be degraded by matrix metalloproteinase-1 (MMP-1), also known as collagenase, and resulting in skin aging^32^. Additionally, elastin fibers comprising about 5% of the dermis layer provide elasticity and resilience of skin. The cleavage of elastin fibers by elastase leads to sagging and wrinkling skin^19,32^. Therefore, the bioactive compounds with anti-collagenase and anti-elastase properties could delay skin aging process.

**Figure 6:**
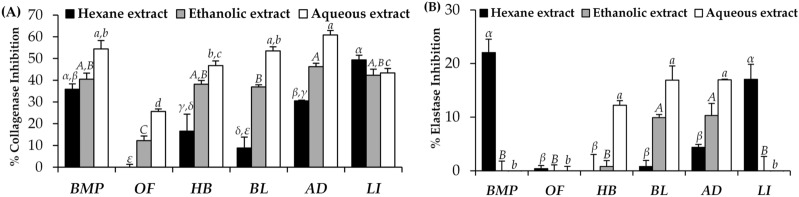
Anti-aging activities of Thai edible insect extracts, including *B. mori* (BM), *O. fuscidentalis* (OF), *Euconocephalus* sp. (EU), *P. succincta* (PS), *A. domesticus* (AD), and *L. indicus* (LI)investigated by collagenase inhibitory determination **(A)** and elastase inhibitory determination **(B)**. The data are expressed as mean ± SD (n = 3). The Greek alphabet letters (*α, β, γ, δ*, and *ε*) indicate significant differences among hexane extracts, the capital letters (*A, B,* and *C*) indicate significant differences among ethanolic extracts, the small case letters (*a, b, c,* and *d*) indicate significant differences among aqueous extracts. The data were analyzed using One-Way ANOVA followed by post hoc Tukey test (*p* < 0.05).

The anti-aging activity of each insect extract is shown in Fig. 6. Among various solvents, aqueous extracts of most insects possessed the highest anti-collagenase activities, followed by ethanolic extracts, and hexane extracts, respectively. Interestingly, the aqueous extract of *A. domesticus*, *B. mori*, and *P. succincta* possessed the significantly highest anti-collagenase activities with the collagenase inhibition of 60.8 ± 2.1%, 54.4 ± 3.9%, and 53.5 ± 1.9%, respectively (*p* < 0.05). These findings supported a previous study reported that the extract of *Gryllus bimaculatus* De Geer, which is one of cricket species in Gryllidae family, had a protective effect against wrinkle formation via collagen degradation inhibition^33^.

Besides, the significantly highest elastase inhibitory activities were found in the aqueous extract of *A. domesticus* (17.0 ± 0.1%), *P. succincta* (16.9 ± 2.7%), and *Euconocephalus* sp. (12.2 ± 0.9%), as well as hexane extracts of *B. mori* (22.1 ± 2.5%) and *L. indicus* (17.1 ± 2.8%) (*p* < 0.05). These results were in good accordance with a previous study reported that *A. domesticus* possessed strong pancreatic elastase inhibitory activity^34^.

### Irritation properties of insect extracts

The irritation properties of Thai edible insect extracts were investigated using HET-CAM assay which had been verified for reliability. In the present study, no irritation sign on CAM was induced by a negative control (0.9% w/v NaCl solution) and vehicle controls (DI water and 0.05% w/v DMSO).

**Figure 7:**
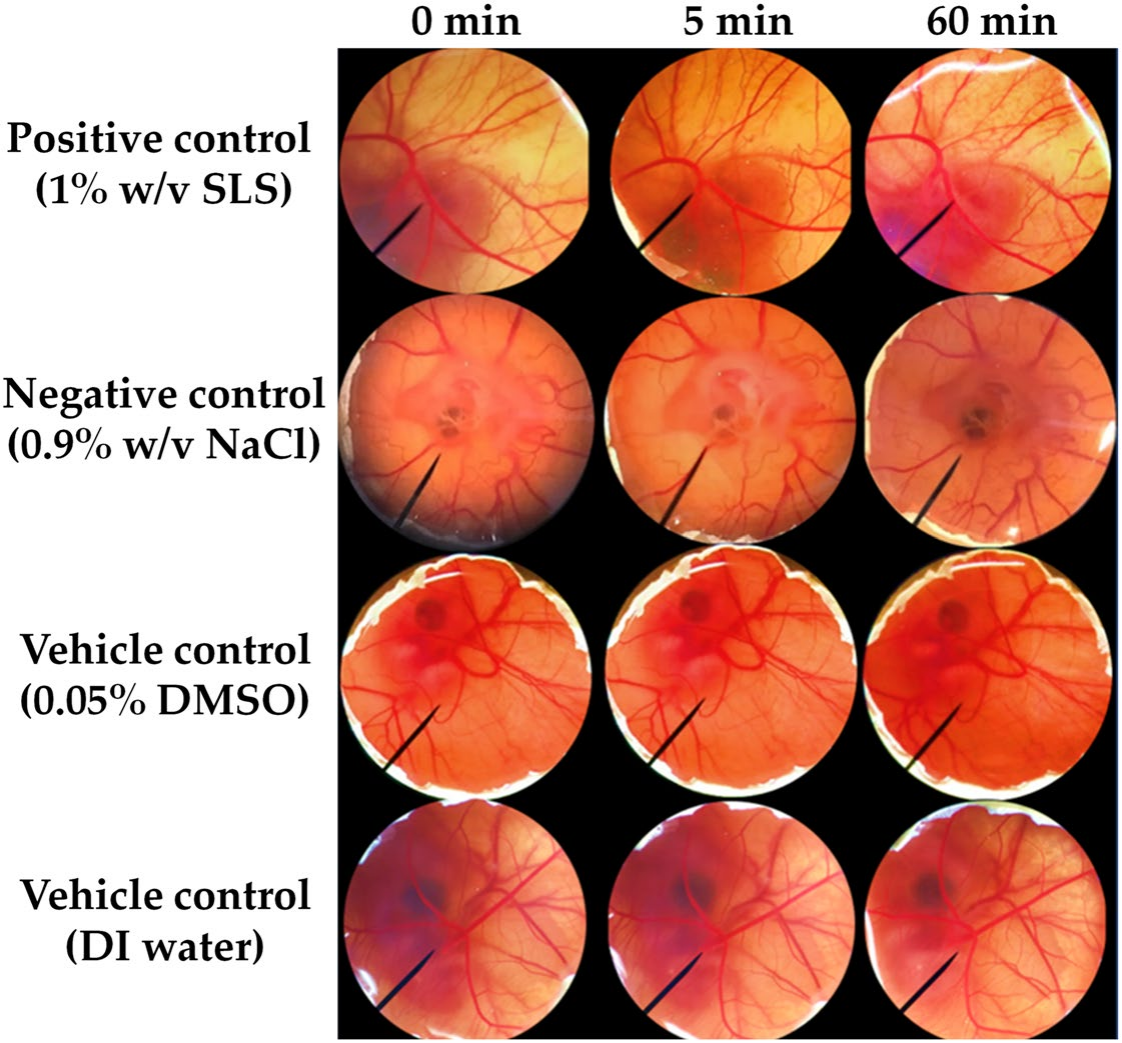
Effect of 1% w/v SLS (positive control), 0.9% w/v NaCl solution, 0.05% DMSO (vehicle control), and DI water (vehicle control) on chorioallantoic membrane before exposure (0 min), after 5 min, and 60 min exposure.

In contrast, severe irritation signs on the CAM were detected in a positive control (1% w/v SLS) with IS score of 10.0 ± 0.5 (Table 1). All signs of irritation, including hemorrhage, coagulation, and vascular lysis were detected on the CAM exposed to 1% w/v SLS within 5 min and more pronounced after 60 min as shown in Fig. 7.

**Table 1:** Irritation score (IS) and irritation levels from HET-CAM assay (n = 3).

Sample	IS	Irritation level
Positive control (1% w/v SLS)	10.0 ± 0.5	Severe irritation
Negative control (0.9% w/v NaCl)	0.0 ± 0.0	No irritation
Vehicle (0.05% w/v DMSO)	0.0 ± 0.0	No irritation
Vehicle (DI water)	0.0 ± 0.0	No irritation

**Figure 8:**
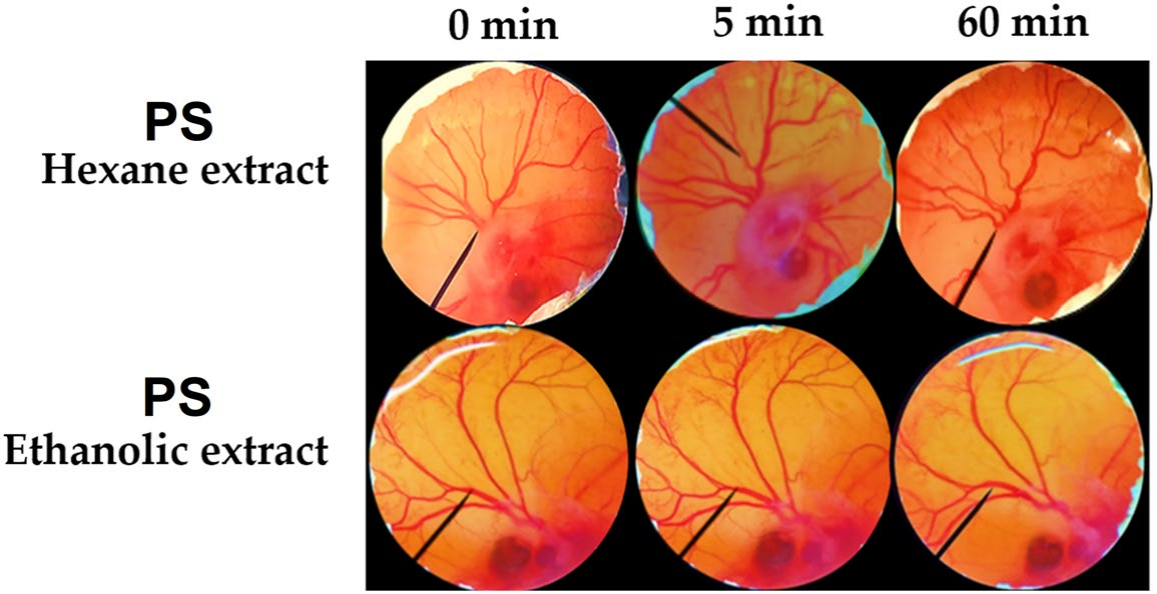
Effect of hexane and ethanolic extracts of *P. succincta* (PS) on chorioallantoic membrane before exposure (0 min), after 5 min, and 60 min exposure.

All insect extracts in the present study were safe since they induced no irritation on CAM, except for *P. succincta*. Ethanolic and hexane extracts of *P. succincta* induced moderate irritation with IS of 6.2 ± 0.5 and 7.4 ± 0.4, respectively. All irritation signs were detected on the CAM after exposed to *P. succincta* hexane extract, whereas only vascular lysis and hemorrhage were detected on the CAM after exposed to the *P. succincta* ethanolic extract as shown in Fig. 8. The results were related well to a previous study reported that *P. succincta* could trigger hypersensitivity reaction and *P. succincta* was identified as a cross-reacting allergen with crustaceans^35^. Therefore, the hexane and ethanolic extracts of *P. succincta* might be noted as a skin irritant and had a precaution for using topically on human skin.

Since HET-CAM assay was usually employed as an alternative irritation test for human tissue, including eye and/or skin irritation^36^. Extracts from *B. mori*, *O. fuscidentalis*, *Euconocephalus* sp., *A. domesticus*, and *L. indicus* were suggested as safe for using topically on human skin.

### Skin irritation effects of insect extracts on human volunteers

**Figure 9:**
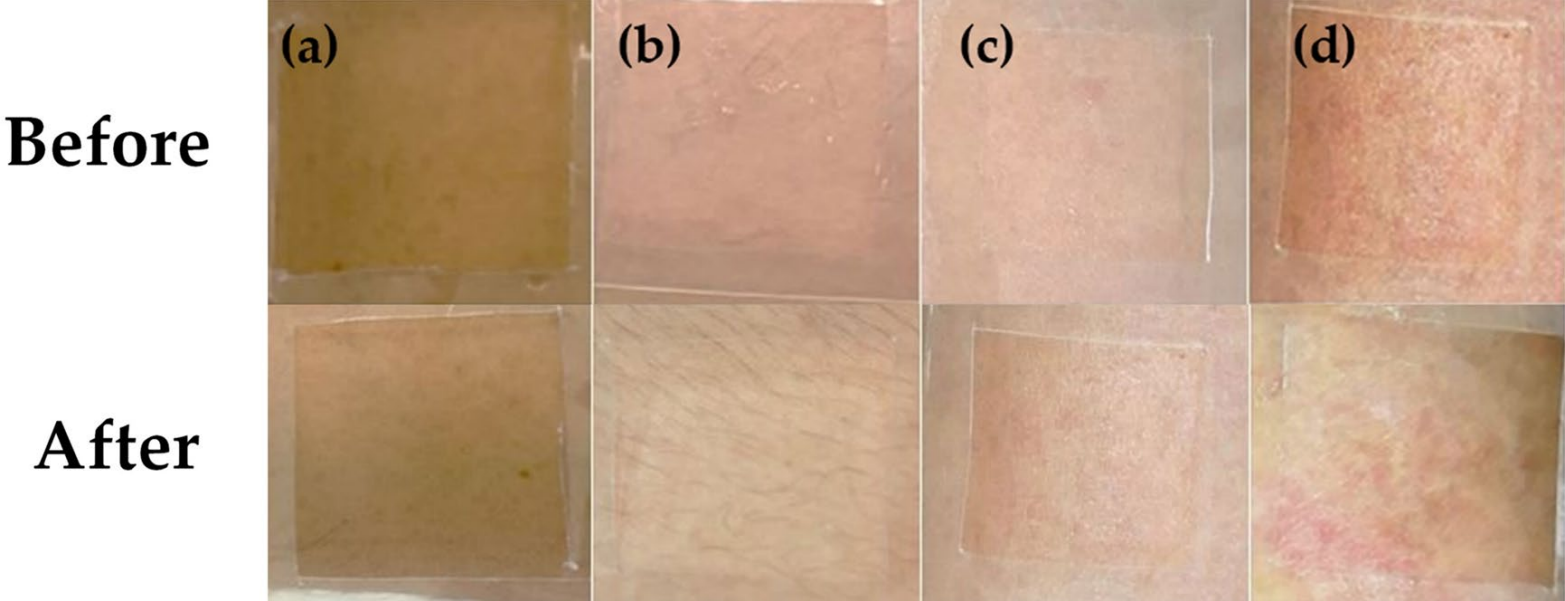
Effect of Thai edible insect extracts on human skin before application (above) and after application (below), including aqueous extract of *B. mori* (BM) (**a**), aqueous extract of *L. indicus* (LI) (**b**), hexane extract of *L. indicus* (LI) (**c**), and ethanolic extract of *B. mori* (BM) (**d**).

The irritation effects of Thai edible insect extracts on human skin are shown in Fig. 9. Since the ethanolic and hexane extracts of *P. succincta* induced irritation signs in HET-CAM test, they were excluded from a clinical study in humans. Among various insects, *B. mori* and *L. indicus* extracts induced skin irritation in humans. The aqueous extracts of *B. mori* and *L. indicus* induced itching in 2 among 30 individual volunteers. Additionally, hexane extract of *L. indicus* induced mild erythema, whereas ethanolic extract of *B. mori* showed severe signs of skin irritation, including papules and itching. However, there were no signs of edema, vesicles, bullae, or weeping in any volunteer. These results related well with a previous study reported that tropomyosin, which was a dimeric coiled-coil protein for muscle regulation, might contribute significantly to *B. mori* allergy. Furthermore, *B. mori* has been reported to be one of a strong IgE cross-reactivity with crustacean^37^. Additionally, a previous study also reported that *L. indicus* appeared to be the insect that was most likely to cause an allergic reaction^38^. Nonetheless, there have not been identified allergen of *L. indicus* in previous studies. Thus, the extracts of *B. mori* and *L. indicus*, which caused irritation in human volunteers, should avoid for further topical applications.

In conclusion, there was a significant difference among different insect species (Wilks’ lambda = 0.000, F (45,128.3) = 350.975, *p* = 0.000) and extraction method (Wilks’ lambda = 0.000, F (18,56) = 1164.921, *p* = 0.000). Aqueous extracts of *A. domesticus* exhibited the significantly highest antioxidant, anti-collagenase, and anti-elastase activities without irritation in both HET-CAM and clinical studies. As a result, *A. domesticus* was proposed for further usage as a cosmeceutical active ingredient for anti-wrinkle skin care. However, it was advised that the chemicals responsible for these biological activities should be further identified. Although utilizing the entire body of the insect would facilitate scale-up production in future applications, further independently investigation for each part of the insect, such as the head, thorax, and abdomen of *A. domesticus* would be suggested. The current study's findings would lead to the selection of an appealing insect family for further intensive comparative research.

## Materials and methods

### Insect material

Frozen *B. mori, O. fuscidentalis, Euconocephalus sp., P. succincta, A. domesticus,* and *L. indicus* were purchased from a local market in Chiang Mai, Thailand during October 2020. Each Thai edible insects were identified by Mr. Pattarawich Dawwrueng, an entomologist at Department of Entomology, Faculty of Agriculture, Kasetsart University. The frozen insects were defrosted overnight at room temperature and dried using a hot air oven at 45 °C until constant weights were obtained. The dried insects were then grounded into fine powders using a Moulinex® blender, model number LM2070BD (Moulinex SA, Bagnolet, France) and kept in a desiccator until further use.

### Chemical reagents

Acetic acid, 2,2′-azinobis (3-ethylbenzothiazoline-6-sulfonic acid) diammonium salt (ABTS), 2,2-diphenyl-1-picrylhydrazyl radical (DPPH), 2, 4, 6 tripyridyl-s-triazine (TPTZ), 6-hydroxy-2,5,7,8-tetramethylchroman-2-carboxylic acid (Trolox), gallic acid, linoleic acid, and hydrochloric acid (HCl) were purchased from Sigma Aldrich (St. Louis, MO, USA). Collagenase from *Clostridium histolyticum*, N-[3-(2-furyl)acryloyl]-Leu-Gly-Pro-Ala (FALGPA), elastase from porcine pancreas, N-succinyl-Ala-Ala-Ala-p-nitroanilide (AAAVPN), disodium hydrogen phosphate (Na_2_HPO_4_), potassium dihydrogen phosphate (KH_2_PO_4_), potassium persulfate (K_2_S_2_O_8_), potassium chloride (KCl), sodium carbonate (Na_2_CO_3_), sodium chloride (NaCl), ferric chloride (FeCl_3_), ferrous sulfate (FeSO_4_), ferrous chloride (FeCl_2_), and ammonium thiocyanate (NH_4_SCN) were purchased from Fisher Chemicals (Loughborough, UK). Bovine serum albumin was purchased from Merck (Darmstadt, Germany). Tricine and tris base were purchased from Bio-Rad Laboratories (Richmond, CA, USA). Sodium acetate trihydrate (CH_3_COONa·3H2O), sodium hydroxide (NaOH), dimethyl sulfoxide (DMSO), and ethanol were analytical grade and purchased from RCI Labscan Co., Ltd. (Bangkok, Thailand).

### Preparation of insect extract

#### Maceration

The dried powders of insects were macerated using hexane or 95% ethanol with the weight ratio of insect powder to solvent of 1:5 at room temperature for 24 h for 3 cycles. After maceration, the solvents were removed under vacuum at 50 °C using a rotary evaporator (Eyela, Tokyo, Japan) until constant weights were attained. The insect extracts were kept at 4 °C until further use.

#### Digestion

The dried powders of insects were digested using DI water with the weight ratio of insect powder to solvent of 1:3 at 45 °C for 3 h. After digestion, the solvent was eliminated using a freeze dryer (FreeZone 4.5 model 7,750,031, Labconco, Kansas, MO, USA) until constant weights were attained. The insect extracts were kept at 4 °C until further use.

### Determination of protein content of insect extracts

The protein content of insect extracts was investigated using bicinchoninic acid (BCA) assay^39^. Briefly, BCA solution was prepared by mixing sodium carbonate, sodium bicarbonate, bicinchoninic acid, and sodium tartrate in 0.1 M sodium hydroxide solution (final pH 11.25) with 4% w/v aqueous cupric sulfate solution. Then 25 µl of each insect extract solution was mixed with 200 µl of BCA solution and incubated at 37 °C for 2 h. The intense purple color formed by the above reaction was then measured at 562 nm using a multimode detector (BMG Labtech, Ortenberg, Germany). The protein content of each sample was determined by comparison with protein standard of BSA. The results were reported as percentages of protein content comparing to the weight of each extract.

### Determination of antioxidant activities of insect extracts

#### 2,2-azinobis (3-ethylbenzothiazoline-6-sulphonic acid) (ABTS) assay

The free radical scavenging activities against ABTS radical cations (ABTS^·+^) of insect extracts were investigated using ABTS assay^40,41^. Briefly, ABTS^·+^ solution was prepared by mixing 7 mM ABTS solution and 2.45 mM K_2_S_2_O_8_ solution in a volume ratio of 2:3. The mixture was kept in a dark place for 16 h. The ABTS^·+^ solution was then diluted with 20-fold excess of ethanol to obtain an absorbance of 0.7 ± 0.1 units at 750 nm. Consequently, 20 µl of 1 mg/ml of each insect extract solution was mixed with 180 µl of ABTS^·+^ solution and incubated at room temperature for 5 min. Then the mixture was measured for an absorbance at 750 nm using a multimode detector (BMG Labtech, Ortenberg, Germany). The free radical scavenging activity was reported as Trolox equivalent antioxidant capacity (TEAC) which represents the amount of Trolox that is equivalent to 1 mg of each insect extract. The experiments were performed in triplicate.

#### 2,2-diphenyl-1-picrylhydrazyl (DPPH) assay

The free radical scavenging activities against DPPH radical (DPPH^·^) of insect extracts were investigated using DPPH assay^41,42^. Briefly, 20 µl of 1 mg/ml of each insect extract solution was mixed with 180 µl of DPPH^·^ solution in methanol. Following incubation in a dark place for 30 min, the mixture was then measured for an absorbance at 520 nm using a multimode detector (BMG Labtech, Ortenberg, Germany). The scavenging activity was reported as percentage of DPPH^·^ inhibition which was calculated using the following equation: % Inhibition = [(A − B)/A] × 100, where A is the absorbance of the mixture solution containing DPPH^·^ solution and solvent and B is the absorbance of the mixture solution containing DPPH^·^ solution and insect extracts. Ascorbic acid was used as a positive control. The experiments were performed in triplicate.

#### Ferric reducing antioxidant power (FRAP) assay

The ferric ion reducing abilities of insect extracts were investigated using FRAP assay^41–43^. Briefly, FRAP solution was prepared by mixing 5 ml of 10 mM TPTZ solution in 40 mM HCl and 5 ml of 20 mM FeCl_3_ in 50 ml of 0.3 M acetate buffer (pH 3.6). Consequently, 20 µl of 1 mg/ml of each insect extract solution was mixed with 180 µl of FRAP solution and incubated at room temperature for 5 min. The mixture was then measured for an absorbance at 595 nm using a multimode detector (BMG Labtech, Ortenberg, Germany). Ferrous sulfate (FeSO_4_) was used as a standard. The ferric reducing ability was reported as equivalent concentration value (EC_1_), which represents the amount of FeSO_4_ that is equivalent to 1 mg of each insect extract. The experiments were performed in triplicate.

#### Inhibition of lipid peroxidation by ferric thiocyanate (FTC) assay

The lipid peroxidation inhibition activities of insect extracts were investigated by FTC assay with some modifications^41–43^. Briefly, 50 µl of 1 mg/ml of each insect extract solution was mixed with 50 µl of 50% linoleic acid, 50 µl of 10% NH_4_SCN solution, and 50 µl of 2 mM FeCl_2_ solution. The mixture was incubated at 37 °C for 1 h and then measured for an absorbance at 500 nm using a multimode detector (BMG Labtech, Ortenberg, Germany). The result was reported as percentage of lipid peroxidation inhibition which was calculated using the following equation: % Inhibition = {[(C − D) − (A − B)]/(C − D)} × 100, where A is an absorbance of the mixture containing insect extract, linoleic acid, NH_4_SCN, and FeCl_2_ solution, B is an absorbance of the mixture containing insect extract and solvent, C is an absorbance of the mixture containing linoleic acid, NH_4_SCN, and FeCl_2_ solution, and D is an absorbance of the mixture containing only solvent. The experiments were performed in triplicate.

### Determination of anti-ageing activities of insect extracts

#### Anti-collagenase activity by spectrophotometric assay

Anti-collagenase activities of insect extracts were investigated by spectrophotometric assay with some modification^44^. The collagenase enzyme activity was deter-mined before performing the experiment and only more than 90% enzyme activity was used in the experiment. Briefly, 10 µl of 1 mg/ml of each insect extract solution was mixed with 5 unit/ml collagenase enzyme and then incubated at room temperature for 15 min. The solution of 2 mM FALGPA solution and tricine buffer (pH 7.5) were added, respectively. The mixture was immediately measured for an absorbance at 340 nm and continuously measured for 20 min using a multimode detector (BMG Labtech, Ortenberg, Germany). The result was reported as percentage of collagenase inhibition which was calculated using the following equation: % Inhibition = [1 − (A/B)] × 100, where A is the reaction rate of the mixture containing each insect extract, collagenase enzyme, tricine buffer, and FALGPA solution, and B is the reaction rate of the mixture containing collagenase enzyme, tricine buffer, and FALGPA solution. Oleanolic acid was used as a positive control. The experiments were performed in duplicate.

#### Anti-elastase activity determination

Anti-elastase activities of insect extracts were investigated by spectrophotometric assay with some modification^44^. The elastase enzyme activity was determined be-fore performing the experiment and only more than 90% enzyme activity was used in the experiment. Briefly, 10 µl of 1 mg/ml of each insect extract solution was mixed with 4.5 unit/l elastase enzyme and then incubated at room temperature for 15 min. The solution of 1.6 mM AAAPVN and 0.2 mM Tris–HCl buffer (pH 8.0) were added, respectively. The mixture was immediately measured for an absorbance at 410 nm and continuously measured for 20 min using a multimode detector (BMG Labtech, Ortenberg, Germany). The result was reported as percentage of elastase inhibition which was calculated using the following equation: % Inhibition = [1 − (A/B)] × 100, where A is the reaction rate of the mixture containing each insect extract, elastase enzyme, tris HCl buffer, and AAAVPN solution and b is the reaction rate of the mixture containing elastase enzyme, tris HCl buffer, and AAAVPN solution. EGCG was used as a positive control. The experiments were performed in duplicate.

### Determination of irritation properties by Hen’s Egg Test-Chorioallantoic Membrane (HET-CAM) assay

The irritation properties of insect extracts were investigated using HET-CAM assay with some modification^45^. Briefly, the fertilized hen’s eggs were incubated in an automatic hatching machine at 37 ± 0.5 °C and 55 ± 7% of relative humidity for 7 days. Before the investigation, the air chamber was indicated by flooding the light and then the eggshell was opened using an electric drill. Consequently, the white inner membrane, which attached the chorioallantoic membranes (CAM), was moistened with 0.9% w/v NaCl solution and carefully removed using forceps. After that, 30 μl of 1 mg/ml of each insect extract solution was added to the CAM. The irritation signs, including hemorrhage, vascular lysis, and coagulation, were continuously monitored for 5 min under a stereomicroscope (Olympus, Tokyo, Japan). The solution of SLS (1% w/v) and normal saline solution (0.9% w/v NaCl) were used as positive control and negative control, respectively. The irritation property was reported as irritation score which was calculated using the following equation: IS = [((301 − H))/300 × 5] + [((301 − L))/300 × 7] + [((301 − C))/300 × 9], where H is the time of vascular hemorrhage observed, L is the time of vascular lysis observed, and C is the time of vascular coagulation observed. The IS scores are graded as 0.0–0.9 indicates no irritation, 1.0–4.9 indicates mild irritation, 5.0–8.9 indicates moderate irritation, and 9.0–21.0 indicates severe irritation. The irritation signs on the CAM were observed again after 60 min for long term irritation. The experiments were performed in duplicate.

### Clinical irritation test using human patch test

Skin irritation induced by insect extracts was investigated by human patch test with some modifications^46,47^. The experiment was approved by the Ethical Review Committee, Faculty of Pharmacy, Chiang Mai University regarding the Declaration of Helsinki. The approved ethical number was 004/2018, which was valid from 06/02/2018 to 05/02/2019. The clinical trial has been reviewed and approved by Thai Clinical Trials Registry (TCTR) Committee on 17/08/2021. The accessible TCTR identification number is TCTR 20210817002 and the valid URL of the registry is https://www.thaiclinicaltrials.org/show/TCTR 20210817002). Thirty healthy volunteers (11 men and 19 women), aged between 21 and 54 years, were included in the study. Informed consent for study participation was obtained from all volunteers. Briefly, 10 µl of each insect extract was applied on the forearm of each volunteer in the area of 1.5 × 1.5 cm2 using a small spatula. The irritation signs were investigated up to 4 h after application by a qualified person. The skin area with no application of insect extract was used as a negative control. Any volunteer exhibiting unequivocal erythema, papule, pustule, or nodule was considered as a positive result^43^.

### Statistical analysis

Data were reported as mean ± standard deviation (S.D.) from three independently performed experiments with three replicates within each experiment. Statistical analysis for the overall significance was carried out using multivariate analysis of variance (MANOVA) and Wilks' Lambda statistics, followed by one-way analysis of variance (ANOVA) and post hoc Tukey’s post-hoc test for each dependent variable using SPSS program software version 17. Statistically significant difference was denoted when *p* < 0.05.
